# Feeding Strategy to Use Beef Tallow and Modify Farmed Tiger Puffer Fatty Acid Composition

**DOI:** 10.3390/ani13193037

**Published:** 2023-09-27

**Authors:** Feiran Zhang, Lin Li, Xiaoxue Meng, Jian Liu, Xishuai Cui, Qiang Ma, Yuliang Wei, Mengqing Liang, Houguo Xu, Artur Rombenso

**Affiliations:** 1 Yellow Sea Fisheries Research Institute, Chinese Academy of Fishery Sciences, 106 Nanjing Road, Qingdao 266071, China; zfr15981947065@163.com (F.Z.); lilinlilin54546@163.com (L.L.); mengxx127@163.com (X.M.); liujian@ysfri.ac.cn (J.L.); 17863653818@139.com (X.C.); maqiang@ysfri.ac.cn (Q.M.); weiyl@ysfri.ac.cn (Y.W.); liangmq@ysfri.ac.cn (M.L.); 2 Laboratory for Marine Fisheries Science and Food Production Processes, Laoshan Laboratory, 1 Wenhai Road, Qingdao 266237, China; 3 CSIRO, Livestock and Aquaculture Program, Animal Nutrition, Bribie Island Research Centre, Woorim 4507, Australia; artur.rombenso@csiro.au

**Keywords:** Fish farming, *Takifugu rubripes*, omega-3 sparing effect, alternate feeding, dietary regulation

## Abstract

**Simple Summary:**

The supply of fish oil, which provides long-chain polyunsaturated fatty acids (LC-PUFA) but is mostly wild-caught, is stagnating, resulting in limited availability and high prices. Therefore, many alternative lipid sources, mainly vegetable oils and rendered animal fats, are increasingly being applied in fish feeds. However, these lipid sources do not contain LC-PUFA, and therefore lower the fillet quality in terms of the LC-PUFA content. Feeding strategies such as alternate feeding between fish oil- and alternative oils-based diets have been applied to maintain the n-3 LC-PUFA contents in farmed fish. The present study validated the relevant efficacy.

**Abstract:**

A 12-week feeding experiment was conducted to evaluate the effects of replacing fish oil (FO) with beef tallow (BT) on the fatty acid composition of farmed tiger puffer (*Takifugu rubripes*). Two replacement strategies were used: a standard Graded Dietary Replacement of FO with BT (GDR strategy) and Alternate Feeding between FO- and BT-based Diets (AFD strategy). The positive and negative control diets were formulated with 6% FO (FO-C group) or BT (BT-C group) as the sole added lipid source. In the GDR strategy, three experimental diets were formulated, with 25, 50 and 75% of the added FO in the FO-C diet replaced with BT, named 25BT, 50BT and 75BT, respectively. In the AFD strategy, alternated feeding patterns between the FO-C and BT-C diet—namely, 1, 2 and 3 weeks with BT-C followed by 1 week feeding with FO-C (1BT-1FO, 2BT-1FO and 3BT-1FO, respectively)—were applied. Each diet or feeding strategy was assigned to triplicate tanks. The results showed that dietary BT inclusion reduced the contents of long-chain polyunsaturated fatty acids (LC-PUFA) in both the muscle and liver (edible tissues for this species) of the experimental fish, and the liver displayed a more drastic decrease than the muscle. The LC-PUFA content linearly decreased with the decreasing dietary FO levels in the GDR strategy. However, in the AFD strategy, a linear relationship was not observed between the LC-PUFA content and the FO feeding duration. The 3BT-1FO treatment resulted in higher LC-PUFA content than 2BT-1FO. When comparing the two strategies with the same final FO administration level—namely, 50BT vs. 1BT-1FO, and in particular, 75BT vs. 3BT-1FO—the AFD strategy resulted in higher LC-PUFA contents in both the muscle and liver than the GDR strategy. In conclusion, when FO was replaced with BT in the diets, alternate feeding between FO- and BT-based diets resulted in a higher LC-PUFA content than the standard direct replacement. Three weeks of feeding with BT-C followed by one week of feeding with FO-C appeared to be a good alternate feeding pattern. This study provided a promising strategy of FO-sparing in fish farming when the LC-PUFA contents were maintained as high as possible.

## 1. Introduction

For human consumption, fish are the main source of n-3 long-chain polyunsaturated fatty acids (LC-PUFA), characterized by docosahexaenoic acid (22:6n-3, DHA) and eicosapentaenoic acid (20:5n-3, EPA). With the rapid expansion of aquaculture, farmed fish products have become a larger contributor to food fish intake than wild-caught fish products [1]. However, for fish feed production, the fish oil (FO) supply, which provides LC-PUFA but is based on wild-caught fishery, is stagnating. This results in limited availability and high prices of FO [2,3]. Therefore, many alternative lipid sources, mainly vegetable oils and rendered animal fats, are increasingly being applied in fish feeds [4,5]. Because most of the alternative lipid sources do not contain LC-PUFA, the use of them in fish feeds results in an inevitable decrease in LC-PUFA in fish products [6,7]. Feeding strategies have been applied to recover or maintain the n-3 LC-PUFA contents in farmed fish [8,9,10]. The FO-finishing strategy—namely, a FO-based diet is fed during the final production phase after long-term feeding of low-FO diets,—has been widely used in fish farming [10,11,12,13,14,15,16,17,18]. In contrast, however, there has been little information about the efficacy of another feeding strategy; namely, alternate feeding between FO- and alternative oils-based diets [9,19,20,21,22,23]. The limited studies in this area suggest that this alternate feeding strategy can also have high efficiency in maintaining n-3 LC-PUFA levels in farmed fish [9,20,21,22,23]. However, the results were not consistent across species or among different alternate-feeding patterns [19,20,21,22]. More studies are needed to screen the best alternated pattern for a specific fish species.

Among the alternative lipid sources, beef tallow (BT) has been reported to be a good substitute for FO in fish feeds. Beef tallow is rich in saturated fatty acids (SFA) and monounsaturated fatty acids (MUFA), which are preferentially oxidized for energy production in fish [24,25]. Balanced SFA and MUFA profiles in the feed limit the extent of n-3 LC-PUFA β-oxidation as well as the metabolic energy required for lipogenesis processes, and consequently increase the amount of n-3 LC-PUFA retained in farmed fish. This is called the “n-3 LC-PUFA sparing effect” [26]; the “n-3 LC-PUFA sparing effect” is of significance in the consideration of the judicious use of valuable LC-PUFA sources in fish feed [27,28,29,30,31,32,33]. Specifically, BT performed well in exerting the “LC-PUFA sparing effect”. This has been demonstrated in several species, such as Japanese sea bass (*Lateolabrax japonicus*), cobia (*Rachycentron canadum*), Florida pompano (*Trachinotus carolinus*), totoaba (*Totoaba macdonaldi*), Atlantic salmon (*Salmo salar*) and rainbow trout (*Oncorhynchus mykiss*), not only in terms of the LC-PUFA content [34,35,36,37,38], but also the growth performance [33,34,35,36,37,38,39,40,41].

Tiger puffer (*Takifugu rubripes*) is a traditional and important aquaculture species in Asia [42,43,44,45]. After decades of farming, tetrodotoxin has been found in trace or even non-detectable concentrations in farmed tiger puffer, probably primarily due to the use of non-toxic feeds [46]. This further accelerates the farming and marketing of this species. Tiger puffer are very lean, with the muscle lipid content of only around 1% [47]. In lean fish, polar lipids usually account for a large proportion of muscle lipid [48], and thus LC-PUFA are more readily deposited in the muscle [25,49]. In this consideration of this, the LC-PUFA-retaining or -recovering strategies could have high efficiency in this species. This has been evidenced in a recent study on the application of FO-finishing strategy in this species [10], where BT performed the best among several terrestrially sourced oils, including linseed oil, soybean oil, rapeseed oil and palm oil. As a following-up study, the present study aimed to investigate the efficacy of alternate feeding between FO- and BT-based diets in maintaining LC-PUFA, particularly compared to the direct graded FO replacement in fish diets.

## 2. Materials and Methods

### 2.1. Experiment Design and Diets

**Figure 1:**
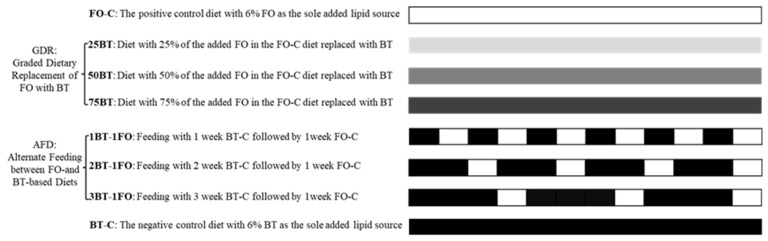
Flow-chart of the feeding trial.

Fish were fed with one of the following 8 feeding regimes (treatments): 2 control groups; 3 treatments of Graded Dietary Replacement of FO with BT (GDR strategy); 3 treatments of Alternate Feeding between FO- and BT-based Diets (AFD strategy) (Figure 1). The positive and negative control diets were formulated with 6% FO (FO-C group) or BT (the BT-C group) as the sole added lipid source, respectively (Table 1). In the GDR strategy, 3 more of the added FO in the FO-C diet replaced with BT, named as 25BT, 50BT and 75BT, respectively. In the AFD strategy, different alternation patterns between the FO-C and BT-C diets—namely, 1, 2 and 3 weeks feeding with BT-C followed by 1 week feeding with FO-C (1BT-1FO, 2BT-1FO and 3BT-1FO, respectively)—were applied (Figure 1). The fatty acid compositions of the experimental diets are presented in Table 2 and Supplementary Figure S1.

### 2.2. Experimental Fish, Feeding Procedure and Sampling

Juvenile tiger puffers (average initial body weight, 12.0 g) were purchased from Hongqi Aquaculture Co. Ltd. (Rizhao, China), and transported to Haiyang Aquaculture Co. Ltd. (Yantai, China), where the feeding experiment was conducted. In order to prevent cannibalism, the fish teeth were cut shorter. The fish were fed a commercial diet for 14 days (3 times a day, at 2% of body weight) during acclimation. After acclimatization, the fish were fasted for 24 h, weighted and randomly distributed into 24 tanks (200 L) at a stocking density of 35 fish per tank. Each feeding regime was assigned to triplicate tanks. Fish were hand-fed to apparent satiation 3 times a day (6:00, 12:00 and 18:30). The tanks were cleaned daily by siphoning out the feces and residual feeds. In the first 2 weeks of the feeding trial, sand-filtered seawater was used. However, an outbreak of harmful algae *Enteromorpha proliferate* in the near sea caused an instantaneous mortality (about half of the experimental fish). After that, we immediately switched the water source to deep-well saltwater. The remaining fish were in good health status in the following days (about 10 weeks). Therefore, the feeding trial continued to the end of week 12. The water quality parameters were monitored weekly: average temperature, 19–21 °C; salinity, 28–30; dissolved oxygen, >6.08 mg L^−1^; pH, 7.59–7.84.

Sampling was conducted at the end of the feeding trial. Before sampling, the fish first were fasted for 24 h. After anesthetized with eugenol (1/10,000 water, *v*/*v*), the fish were individually counted and bulk weighed. After that, from each tank, two fish were randomly selected, and the body length and weight, as well as the viscera and liver weight, were recorded to calculate the condition factor (CF = body weight/body length^3^ × 100), hepatosomatic index (HSI (%) = wet liver weight/fish body weight × 100) and viscerosomatic index (VSI (%) = wet viscera weight/fish body weight × 100). Then, these two fish were used for the analysis of whole-body proximate composition. Four more randomly selected fish per tank were dissected and the tissue samples, including muscle, liver and intestine, were collected for subsequent potential use. All collected samples were immediately frozen with liquid nitrogen, and stored at -76 °C before use. All sampling protocols and fish handling practices of this study were reviewed and approved by the Animal Care and Use Committee of Yellow Sea Fisheries Research Institute.

### 2.3. Assay of Proximate Composition and Fatty Acids

The proximate composition analysis of fish tissues was conducted according to the methods of the Association of Official Analytical Chemists [50]. Briefly, the protein content: Kjeldahl method by measuring total nitrogen (N× 6.25); total lipid: Soxhlet method (petroleum ether extraction); moisture content: oven-drying at 105 °C to constant weight; ash: incineration at 550 °C.

The fatty acid compositions of all tissues (muscle, liver and intestine) were analyzed through gas chromatography (GC-2010 pro, Shimadzu, Japan). Briefly, fatty acids in lyophilized tissue samples were esterified with KOH-methanol and HCL-methanol in 72 °C water bath. Fatty acid methyl esters were extracted with hexane and then analyzed through gas chromatography using a flame ionization detector and a fused silica capillary column (SH-RT-2560, 100 m × 0.25 mm × 0.20 μm). The column temperature increase was programmed: 150 °C to 200 °C at 15 °C min-1; then from 200 °C to 250 °C at 2 °C min-1. The injector and detector temperatures were 250 °C. Fatty acid results were expressed as percentage of each fatty acid with respect to total fatty acids (% TFA).

### 2.4. Quantitative Real-Time Polymerase Chain Reaction (qRT-PCR)

The measurement of the mitochondrial DNA (mtDNA) copy number in muscle and liver, as well as the hepatic expression of lipid metabolism genes, were conducted through qRT-PCR analysis.

For the measurement of mtDNA, DNA in fish tissues was extracted using a TIANamp Genomic DNA Kit (TIANGEN BIOTHECH, China). For the measurement of lipid metabolism genes, total RNA in the liver was extracted using RNAiso Plus (TaKaRa (Dalian), Dalian, China) and reverse transcribed using the PrimeScript™ RT reagent Kit with gDNA Eraser (TaKaRa), according to the user manual.

The primers used are presented in Supplementary Table S1. The 16S rRNA and cytochrome B (CYTB) were used as the two representative genes for mtDNA. The β-actin and EF1α (geometrical mean of Ct value) were used as the reference genes.

The qRT-PCR system was based on 5 μL SYBR Green Pro Taq HS Premix II, 3.2 μL sterilized water, 1 μL cDNA template and 0.4 μL forward primer and reverse primer (10 μM). The thermal cycling conditions consisted of an initial denaturing step at 95 °C for 30 s, 40 cycles of “95 °C for 5 s, 57 °C for 30 s and 72 °C for 30 s”, and a final melting curve from 65 °C to 97 °C (6.4 °C increment/min). The relative gene expression levels were expressed according to the 2^-ΔΔCt^ method [51].

### 2.5. Lipid Metabolism-Related Biochemical Parameters in the Serum

The concentration of total cholesterol (TC), total triglyceride (TG), total bile acid (TBA), high-density lipoprotein cholesterol (HDL-C) and low-density lipoprotein cholesterol (LDL-C) in serum was measured using commercial kits supplied by the Nanjing Jiancheng Bioengineering Institute (Nanjing, China).

### 2.6. Statistical Methods

All data were analyzed using one-way analysis of variance (ANOVA) (SPSS 16.0 for Windows). Before analysis, all percentage data were arcsine transformed. Duncan’s multiple range test was used to detect significant differences between the means. A significant difference was accepted when *p* < 0.05.

## 3. Results

### 3.1. Growth Performance, Proximate Composition and Somatic Indices

Due to an early mortality in the feeding trial, the growth performance was no longer a focus of this study. In general, compared to the FO control group, the other diets resulted in no significant changes in final body weight (Supplementary Table S2).

The final average fish body weight was 73.4 g. With respect to the whole-body proximate composition, the FO replacement with BT reduced the whole-body total lipid content, and the reduction was more severe in the GDR strategy than in the AFD strategy (Table 3). Groups BT-C and 50BT had the lowest whole-body total lipid content, which was significantly (*p* < 0.05) lower than the other groups, with the exception of 75BT. In contrast, the FO replacement with BT affected the whole-body moisture content in an opposite way. The effects of the FO replacement with BT on the total lipid and moisture contents in the liver were similar to the trends described above. No significant difference was observed in the whole-body crude protein content and proximate composition of muscle among the different treatments.

**Figure 2:**
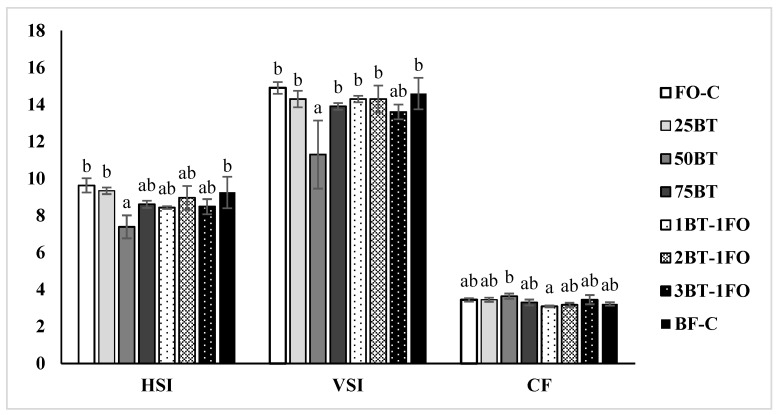
Somatic parameters of juvenile tiger puffer (mean ± standard error). Data bars for a same tissue not sharing a same superscript letter were significantly different (*p* < 0.05).

For the somatic indices, group 50BT showed significantly (*p* < 0.05) lower HSI and VSI than the control groups, but the FO replacement with BT resulted in no significant (*p* > 0.05) changes in the CF compared to the control groups (Figure 2).

### 3.2. Fatty Acid Composition

In general, the dietary BT had a clear effect on the tissue fatty acid composition. In the muscle, the dietary BT significantly reduced the DHA content, but to a lesser extent than the EPA content (Table 4 and Supplementary Figure S2). Accordingly, the dietary BT increased the muscle 18:1n-9 content. Nevertheless, these differences were milder than those across the experimental diets. Although the BT inclusion also increased the 16:0 and 18:2n-6 contents in the diets, these two fatty acids were not proportionally deposited in the muscle.

Similar results were observed in the liver (Table 5 and Supplementary Figure S3). However, compared to the muscle, the BT administration caused a more severe reduction in liver EPA and 18:2n-6. The 16:0 content was consistent among all of the treatments. In the intestine, only marginal differences were displayed in nearly all of the fatty acids (Table 6).

When the GDR and AFD strategies were compared at the same BT administration levels (50BT vs. 1BT-1FO and 75BT vs. 3BT-1FO), in all of the tissues—but particularly the liver—the AFD strategy resulted in higher DHA and EPA contents but lower SFA and MUFA contents than the GDR strategy.

### 3.3. MtDNA Copy Number in the Muscle and Liver

**Figure 3:**
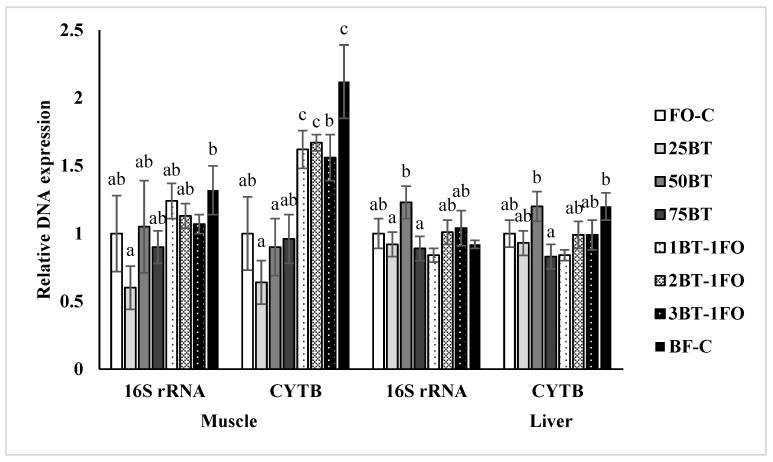
MtDNA copy number in muscle and liver of experimental tiger puffer (mean ± standard error). Data bars for a same gene not sharing a same superscript letter were significantly different (*p* < 0.05). The mtDNA copy number in group FO-C were normalized to be 1.

In general, the expression of the two genes indicated that the mtDNA copy number was similarly different among the different feeding regimes (Figure 3). In the muscle, the BT-C group showed significantly (*p* < 0.05) higher CYTB expression than the FO-C group, and the AFD strategy resulted in higher CYTB expression than the GDR strategy.

### 3.4. Expression of Lipid Metabolism Genes

In general, the dietary BT up-regulated the expression of some peroxisomal fatty acid β-oxidation genes, such as *vlc*, *acox3* and *acaa1* in the liver (Table 7). For genes of glyceride biosynthesis and hydrolysis, compared to the FO-C group, the 3BT-1FO group demonstrated significantly lower expression of *dgat1*, but significantly higher expression of *mgll*. High dietary BT levels down-regulated the expression of *basl*, *pl*, *fabp10a*, *pparα2* and *cyp7a1*. Partial FO replacement with BT upregulated the gene expression of *hmgcr*.

### 3.5. Lipid Metabolism-Related Biochemical Parameters in the Serum

Although significant differences were observed in some of the parameters between some of the groups, there was no significant regression or correlation between the parameters and FO replacement level for both replacement strategies (Supplementary Table S3). The FO replacement with BT tended to reduce the TG concentration in the serum.

## 4. Discussion

The total replacement of FO with BT significantly reduced the DHA content (19.3% to 13.3% of TFA, decreased by 31%) in the muscle. However, a recent study on the same species showed that BT resulted in higher muscle DHA content than linseed oil, soybean oil, rapeseed oil and palm oil when FO was completely replaced [10]. Similar results have also been observed in other marine carnivorous species, such as cobia (*Rachycentron canadum*), Japanese seabass (*Lateolabrax japonicus*) and Atlantic salmon (*Salmo salar*) [38,39,52], although inconsistent results were observed in silvery-black porgy (*Sparidentex hasta*) and large yellow croaker (*Pseudosciaena crocea*) [53,54].

With respect to the partial replacement of FO with BT, the graded dietary replacement linearly decreased the muscle DHA content, but in the alternation feeding strategy, the muscle DHA content was not linearly correlated with the BT administration level. Studies have shown the existence of cyclical mechanisms relative to FA retention/utilization [7,25]. This not only applies to long-term weekly alternation between different diets, but also to circadian alternation [19,20,21,55]. In a long-term alternation between FO- and alternative oil-based diets, compensatory effects on LC-PUFA deposition may exist following FO depletion upon the refeeding of FO-rich diets [4,19,56].

More importantly, the present study is the first to compare the efficacy of two different FO replacement strategies. The results showed that in both comparison sets with the same FO replacement levels—namely, 50BT vs. 1BT-1FO and 75BT vs. 3BT-1FO—the ADF strategy resulted in higher muscle DHA contents than the GDR strategy (the muscle DHA in 3BT-1FO is 108% that of 75BT). This could be related to the compensatory effects mentioned above. On the other hand, it could also be due to the fact that in the AFD strategy, before the final sampling, the fish were fed the FO-based diet for 1 week, which was equal to a miniature FO-finishing period. Nevertheless, if this cyclical miniature FO-finishing period works, it is meaningful in aquaculture practices.

In the liver—which is also an edible tissue for this species—although, in general, similar results were observed, two main differences were observed. Firstly, compared to muscle, the hepatic EPA content was decreased more drastically by dietary BT inclusion. This was partly due to the fact that the liver is a lipid storage organ in tiger puffer [47], and thus EPA was not selectively deposited as it is in the muscle where LC-PUFA mainly function in the maintenance of cellular membrane fluidity [57]. A recent meta-analysis showed that the average EPA content in the intraperitoneal fat of fish was higher than that in the liver [7]. EPA may be deposited over DHA in lipid storage organs as a preferred substrate for energy supply. Previous research on Atlantic salmon revealed that EPA can be extensively β-oxidized when supplied in surplus [58]. Furthermore, when n-3 LC-PUFA were deprived in the diets, the fish phospholipids displayed selective DHA retention but complete EPA depletion [59]. The second difference between the liver and muscle was that it was clear that, in the liver, the AFD strategy resulted in higher LC-PUFA content than the GDR strategy. For 75BT vs. 3BT-1FO in particular, significant differences were observed in the liver LC-PUFA content.

It was hypothesized that mainly SFA and MUFA spared the n-3 LC-PUFA. The differences in the SFA and MUFA contents among the fish tissues of the different groups were indeed smaller than those among the diets. This was apparent for typical SFA and MUFA such as 14:0, 16:0, 16:1n-7 and 18:1n-9. SFA and MUFA are favorable substrates for β-oxidation in the energy production of fish [25,60], and excess SFA and MUFA in BT could be easily β-oxidized. Nevertheless, significant differences in 18:1n-9 in the livers between the FO- and BT-based groups (like FO-C vs. most BT groups) still existed. This may be due to the fact that 18:1n-9 is a primary substrate for triacylglycerol synthesis, and is typically utilized for energy storage [61]. On the other hand, it is worth noting that the utilization of SFA and MUFA for energy production is not uniform across fish species. For instance, a preference and high efficiency for β-oxidation of long-chain MUFA, such as 24:1n-9, 22:1n-11, 22:1n-9 and 20:1n-9, has been observed in salmonids, despite a limited dietary supply [4,12,25]. In contrast, in sunshine bass (*Morone chrysops* ♀ × *M. saxatilis* ♂), SFA were more strongly preferred for catabolism [30]. This study also highlights the importance and need for an understanding of the fate of individual fatty acids in fatty acid groupings in the context of the n-3 LC-PUFA sparing effect.

In order to evaluate the general energy supply status, the mitochondrial DNA copy number in the muscle and liver was assayed in the present study. The lack of differences in mitochondrial DNA copy number indicates a similar energy supply status among the groups, although the mitochondrial DNA copy number was not directly related to a certain fatty acid composition. Alternate feeding between FO- and BT-based diets even tended to increase the mitochondrial DNA copy number in the fish tissues.

In addition to SFA and MUFA, 18:2n-6 was also differently abundant between FO and BT. Previous studies have suggested that tiger puffer may have a low capacity for utilizing n-6 fatty acids [62]. The higher 18:2n-6 retention in the liver when higher levels of BT were included in the diet provided further evidence for this hypothesis. In other species, it has also been reported that the level of 18:2n-6 β-oxidation is typically lower compared to 18:3n-3 [49,56]. Another characteristic fatty acid of BT is 18:0. However, 18:0 is found in low quantities in fish and is a poor substrate for energy mobilization and triacylglycerol synthesis [60]. Nevertheless, the muscle of tiger puffer has a high 18:0 content (around 14% of TFA)—much higher than other fish species [8]. Stearic acid is abundant in polar lipids, but high polar lipid contents in tiger puffer muscle cannot explain the high 18:0 content in the muscle because Atlantic cod (*Gadus morhua*), another species with high polar lipid contents in the muscle, do not have such a high 18:0 content in their muscle [49,63,64,65,66,67]. Instead of being recognized as a less useful fatty acid, 18:0 has been found to be relatively abundant in tissues with specific functions, such as the heart and brain of both fish and mammals [68,69,70]. The potential special functions of 18:0 in tiger puffer remain elusive and warrant further studies.

The intestine is not an edible part. However, previous studies have shown that in tiger puffer, the intestine could be a lipid metabolism center, whereas the liver is more likely a pure lipid storage organ [8]. In this study, little difference was observed in the intestinal fatty acid composition, indicating that the dietary BT induced few changes in the lipid metabolism. This was further evidenced by the gene expression results. Dietary BT rarely affected the gene expression relating to lipid metabolism processes. The fold changes in the expression of most genes caused by dietary BT were lower than 2.

Nevertheless, although the changes in gene expression were relatively mild, some of the changes are still worth discussing. In particular, the inclusion of dietary BT up-regulated the expression of some peroxisomal fatty acid β-oxidation genes, including *vlc*, *acox3* and *acaa1* in the liver, but did not significantly affect the expression of the mitochondria fatty acid β-oxidation gene, *cpt-1*. Previous fish studies have indicated that peroxisomal β-oxidation accounts for a high proportion (30–50%) of total hepatic β-oxidation [71,72], but a lower proportion (20–40%) in the muscle [73]. The present results suggested that the 14–18C fatty acids in BT could be efficiently β-oxidized in the liver. This was consistent with the higher expression of *lipc* (also known as hepatic triacylglycerol lipase), which catalyzed the hydrolysis of triglycerides and phospholipids present in circulating plasma lipoproteins in the liver in the BT groups. In addition, the expression of lipogenic genes *acacβ* and *fas* was not affected by the dietary treatments, indicating that the dietary replacement of FO with BT has limited effects on lipogenesis.

Regarding the genes for glyceride biosynthesis and hydrolysis, compared to the FO-C group, the 3BT-1FO group showed significantly lower expression of *dgat1*, which is a key acyltransferase in triacylglycerol formation [74], but significantly higher expression of *mgll*, which catalyzes the last step of triacylglycerol hydrolysis. This result indicates that in this pattern of alternation between FO- and BT-based diets, triacylglycerol may be better utilized. This was consistent with the superiority of this group in terms of the growth performance.

High dietary BT levels down-regulated the expression of lipases *basl* and *pl*, which non-specifically hydrolyzes triacylglycerol completely to glycerol and free fatty acids, and hydrolyzes triacylglycerol specifically at sn-1,3, respectively [75]. This could be related to the fact that the high 18:0 content in BT is difficult to digest [76]. Regarding cholesterol and bile acid biosynthesis, the partial replacement of FO with BT up-regulated the gene expression of *hmgcr*, which is the rate-limiting enzyme of cholesterol biosynthesis, but down-regulated that of *cyp7a1*, which is the rate-limiting enzyme for bile acid synthesis from cholesterol [77]. This could be due to the fact that the FO had a higher cholesterol content than the BT. Nevertheless, this difference was not reflected in the levels of cholesterol and bile acid in the serum (Supplementary Table S2), indicating the high buffering capacity of fish serum. Among the lipid metabolism-related transcription factors, *srebf1* and *fxr* were the most significantly regulated. These changes could be related to the changes in the gene expression of *hmgcr* and *cyp7a1*, respectively, considering the functions of these two transcription factors in the regulation of cholesterol and bile acid biosynthesis. Other gene expression results, such as the down-regulation of *fabp10a* and *pparα2* and the up-regulation of *lpl* resulting from the dietary BT, were difficult to explain based on the current available information. The precise mechanisms involved need to be elucidated in future studies.

## 5. Conclusions

The dietary inclusion of beef tallow reduced the LC-PUFA content in the muscle and liver of juvenile tiger puffer. However, the n-3 LC-PUFA sparing effect of SFA and MUFA in beef tallow can still be observed, particularly when alternate feeding between fish oil- and beef tallow-based diets was applied. Compared to direct fish oil replacement in the diets, the alternate feeding between fish oil- and beef tallow-based diets resulted in higher n-3 LC-PUFA contents in the fish. Three weeks of feeding with the beef tallow-based diet followed by one week of feeding with the fish oil-based diet seem to be the best feeding strategy to maximize the n-3 LC-PUFA deposition and reduce the reliance on fish oil.

## Figures and Tables

**Table 1:** Composition and nutrition contents of the experimental diets (% dry matter).

Item	FO-C	25BT	50BT	75BT	BT-C
Ingredients					
Fishmeal ^1^	42.0	42.0	42.0	42.0	42.0
Corn gluten meal ^1^	8.00	8.00	8.00	8.00	8.00
Soybean meal ^1^	14.0	14.0	14.0	14.0	14.0
Wheat meal ^1^	20.7	20.7	20.7	20.7	20.7
Brewer’s yeast ^1^	5.00	5.00	5.00	5.00	5.00
Mineral premix ^1^	0.50	0.50	0.50	0.50	0.50
Vitamin premix ^1^	1.00	1.00	1.00	1.00	1.00
Monocalcium phosphate	1.00	1.00	1.00	1.00	1.00
L-ascorbyl-2-polyphosphate	0.20	0.20	0.20	0.20	0.20
Choline chloride	0.20	0.20	0.20	0.20	0.20
Betaine	0.30	0.30	0.30	0.30	0.30
Ethoxyquin	0.02	0.02	0.02	0.02	0.02
Mold inhibitor ^2^	0.10	0.10	0.10	0.10	0.10
Soya lecithin	1.00	1.00	1.00	1.00	1.00
Fish oil ^1^	6.00	4.50	3.00	1.50	0.00
Beef tallow	0.00	1.50	3.00	4.50	6.00
Total	100	100	100	100	100
Proximate composition					
Moisture (%)	5.18	5.40	4.32	4.61	5.33
Crude Protein	46.4	46.4	46.5	46.5	46.0
Total lipid	11.5	11.8	11.5	11.7	11.7
Ash	9.22	9.33	9.17	9.19	9.37

^1^ The crude protein content of fishmeal, corn gluten meal, soybean meal, wheat meal and brewer’s yeast, was 68.95%, 65.38%, 52.20%, 15.10% and 53.66% (dry matter), respectively. The crude lipid content of them was 9.94%, 0.70%, 1.65%, 1.08% and 2.19% (dry matter), respectively. All these ingredients, as well as mineral premix and vitamin premix, which are designed for marine fish, were purchased from Qingdao Surgreen Bioengineering Co., Ltd. (Qingdao, China). ^2^ Contained 50% calcium propionic acid and 50% fumaric acid.

**Table 2:** The fatty acid compositions in the oil sources and experimental diets (%TFA).

Fatty Acid	Fish Oil	Beef Tallow	FO-C	25BT	50BT	75BT	BT-C
14:0	6.44	2.77	6.18	4.91	4.11	3.71	3.33
16:0	22.5	33.2	24.6	25.5	26.1	27.3	28.3
18:0	5.53	20.3	5.44	8.69	12.1	14.5	17.9
∑SFA	34.5	56.3	36.2	39.1	42.3	45.5	49.5
16:1n-7	6.45	1.74	6.89	5.85	5.02	4.27	3.60
18:1n-9	19.3	38.7	17.6	20.5	23.8	25.8	26.8
∑MUFA	25.8	40.5	24.5	26.3	28.8	30.1	30.4
18:2n-6	14.8	2.92	15.7	14.0	11.9	10.6	8.98
18:3n-6	0.12	0.02	0.06	0.17	0.13	0.10	0.08
20:2n-6	0.26	0.02	0.26	0.24	0.22	0.19	0.15
20:4n-6	0.66	0.01	0.79	0.83	0.8	0.67	0.61
∑n-6 PUFA	15.8	2.98	16.8	15.2	13.1	11.6	9.82
18:3n-3	2.04	0.17	1.71	1.58	1.33	0.94	0.75
20:3n-3	0.18	0.05	0.11	0.10	0.10	0.07	0.04
20:5n-3	9.85	0.01	10.81	9.13	7.49	6.39	5.15
22:5n-3	1.00	0.02	1.37	1.28	1.11	0.96	0.83
22:6n-3	10.85	0.01	8.49	7.24	5.76	4.45	3.42
∑n-3 PUFA	23.9	0.26	22.5	19.3	15.8	12.8	10.2
DHA/EPA	1.10	0.69	0.79	0.79	0.77	0.70	0.67

TFA: total fatty acids; SFA: saturated fatty acid; MFUA: monounsaturated fatty acid; PUFA: polyunsaturated fatty acid.

**Table 3:** Tissue proximate composition of experimental tiger puffer (mean ± standard error).

Parameters	FO-C	GDR			AFG			BT-C
		25BT	50BT	75BT	1BT-1FO	2BT-1FO	3BT-1FO	
Whole body								
Crude protein	15.4 ± 0.12	15.6 ± 0.20	15.3 ± 0.29	15.6 ± 0.09	15.6 ± 0.13	15.4 ± 0.15	15.7 ± 0.08	15.5 ± 0.08
Total lipid	6.22 ± 0.12 ^d^	5.78 ± 0.14 ^cd^	4.58 ± 0.24 ^a^	4.85 ± 0.17 ^ab^	6.07 ± 0.35 ^d^	5.31 ± 0.25 ^bc^	5.63 ± 0.17 ^cd^	4.63 ± 0.19 ^a^
Ash	2.30 ± 0.06 ^ab^	2.47 ± 0.04 ^b^	2.35 ± 0.05 ^ab^	2.39 ± 0.02 ^b^	2.27 ± 0.09 ^a^	2.29 ± 0.04 ^a^	2.29 ± 0.06 ^a^	2.30 ± 0.03 ^ab^
Moisture	75.4 ± 0.30 ^a^	75.4 ± 0.17 ^a^	77.4 ± 0.15 ^c^	76.7 ± 0.29 ^bc^	75.3 ± 0.48 ^a^	76.3 ± 0.50 ^ab^	76.0 ± 0.27 ^ab^	76.8 ± 0.20 ^bc^
Muscle								
Crude protein	18.6 ± 0.17	19.4 ± 0.33	19.3 ± 0.48	18.9 ± 0.12	19.5 ± 0.66	18.3 ± 0.40	18.3 ± 0.58	18.9 ± 0.15
Total lipid	1.06 ± 0.02 ^ab^	1.15 ± 0.05 ^b^	1.13 ± 0.03 ^b^	1.05 ± 0.03 ^ab^	1.09 ± 0.13 ^ab^	0.93 ± 0.02 ^a^	0.93 ± 0.03 ^a^	1.04 ± 0.01 ^ab^
Moisture	78.9 ± 0.26 ^ab^	78.0 ± 0.36 ^ab^	78.1 ± 0.52 ^ab^	78.7 ± 0.14 ^ab^	77.9 ± 0.77 ^a^	79.2 ± 0.46 ^ab^	79.4 ± 0.60 ^b^	78.6 ± 0.24 ^ab^
Liver								
Total lipid	66.5 ± 0.73 ^d^	60.8 ± 0.94 ^bc^	60.1 ± 0.90 ^b^	62.9 ± 0.89 ^c^	62.6 ± 0.46 ^c^	60.1 ± 0.79 ^b^	65.4 ± 0.58 ^d^	55.5 ± 0.71 ^a^
Moisture	31.6 ± 1.67 ^ab^	29.0 ± 0.72 ^ab^	33.0 ± 1.30 ^b^	31.6 ± 2.04 ^ab^	28.9 ± 1.08 ^ab^	28.2 ± 0.91 ^a^	27.9 ± 0.67 ^a^	29.0 ± 1.40 ^ab^

Data in a same row sharing same superscript letters are not significantly different (*p* > 0.05).

**Table 4:** Muscle fatty acid composition of experimental tiger puffer (% TFA, mean ± standard error).

Fatty Acid	FO-C	GDR			AFG			BT-C
		25BT	50BT	75BT	1BT-1FO	2BT-1FO	3BT-1FO	
14:0	0.69 ± 0.04 ^c^	0.64 ± 0.03 ^bc^	0.65 ± 0.04 ^bc^	0.55 ± 0.05 ^ab^	0.66 ± 0.01 ^bc^	0.60 ± 0.03 ^abc^	0.57 ± 0.05 ^abc^	0.51 ± 0.02 ^a^
16:0	22.2 ± 0.14 ^b^	22.4 ± 0.37 ^b^	22.3 ± 1.01 ^b^	20.7 ± 0.15 ^a^	21.9 ± 0.12 ^ab^	21.8 ± 0.12 ^ab^	21.9 ± 0.23 ^ab^	20.7 ± 0.08 ^a^
18:0	13.6 ± 0.14 ^a^	13.8 ± 0.15 ^ab^	14.7 ± 0.62 ^b^	14.7 ± 0.45 ^b^	13.8 ± 0.16 ^ab^	14.2 ± 0.32 ^ab^	14.8 ± 0.13 ^b^	14.3 ± 0.08 ^ab^
∑SFA	36.5 ± 0.17 ^ab^	36.8 ± 0.39 ^ab^	37.7 ± 1.64 ^b^	35.9 ± 0.26 ^ab^	36.4 ± 0.29 ^ab^	36.6 ± 0.18 ^ab^	37.2 ± 0.20 ^ab^	35.5 ± 0.12 ^a^
16:1n-7	1.29 ± 0.06 ^b^	1.26 ± 0.04 ^ab^	1.19 ± 0.09 ^ab^	1.05 ± 0.10 ^ab^	1.23 ± 0.05 ^ab^	1.19 ± 0.03 ^ab^	1.15 ± 0.10 ^ab^	1.03 ± 0.10 ^a^
18:1n-9	11.5 ± 0.12 ^a^	12.3 ± 0.12 ^ab^	12.7 ± 0.54 ^bc^	15.0 ± 0.38 ^e^	13.1 ± 0.11 ^bcd^	14.0 ± 0.27 ^d^	13.5 ± 0.29 ^cd^	16.7 ± 0.32 ^f^
∑MUFA	12.8 ± 0.16 ^a^	13.6 ± 0.15 ^ab^	13.9 ± 0.54 ^b^	16.0 ± 0.48 ^d^	14.3 ± 0.06 ^bc^	15.2 ± 0.29 ^cd^	14.6 ± 0.31 ^bc^	17.7 ± 0.40 ^e^
18:2n-6	11.0 ± 0.08 ^ab^	11.0 ± 0.14 ^ab^	10.7 ± 0.48 ^a^	11.7 ± 0.29 ^bc^	11.1 ± 0.04 ^ab^	11.4 ± 0.15 ^ab^	11.1 ± 0.09 ^ab^	12.2 ± 0.31 ^c^
18:3n-6	0.09 ± 0.02 ^bc^	0.10 ± 0.00 ^c^	0.07 ± 0.01 ^ab^	0.06 ± 0.01 ^ab^	0.09 ± 0.00 ^bc^	0.07 ± 0.00 ^ab^	0.08 ± 0.01 ^bc^	0.05 ± 0.00 ^a^
20:2n-6	0.89 ± 0.05 ^c^	0.89 ± 0.02 ^c^	0.85 ± 0.05 ^bc^	0.75 ± 0.01 ^ab^	0.83 ± 0.04 ^ab^	0.83 ± 0.03 ^ab^	0.85 ± 0.03 ^bc^	0.73 ± 0.01 ^a^
20:4n-6	0.12 ± 0.01 ^bcd^	0.15 ± 0.02 ^d^	0.12 ± 0.01 ^bcd^	0.13 ± 0.01 ^cd^	0.10 ± 0.00 ^abc^	0.10 ± 0.01 ^ab^	0.08 ± 0.01 ^a^	0.14 ± 0.00 ^d^
∑n-6 PUFA	12.1 ± 0.09 ^ab^	12.1 ± 0.16 ^ab^	11.7 ± 0.44 ^a^	12.6 ± 0.27 ^bc^	12.1 ± 0.04 ^ab^	12.4 ± 0.13 ^abc^	12.1 ± 0.07 ^ab^	13.2 ± 0.31 ^c^
18:3n-3	0.31 ± 0.01 ^c^	0.26 ± 0.02 ^ab^	0.27 ± 0.02 ^ab^	0.23 ± 0.03 ^a^	0.29 ± 0.01 ^bc^	0.26 ± 0.01 ^ab^	0.24 ± 0.01 ^ab^	0.24 ± 0.01 ^ab^
20:3n-3	2.28 ± 0.07 ^c^	2.28 ± 0.03 ^c^	2.28 ± 0.11 ^c^	2.04 ± 0.07 ^ab^	2.15 ± 0.04 ^bc^	2.10 ± 0.04 ^ab^	2.19 ± 0.01 ^bc^	1.93 ± 0.01 ^a^
20:5n-3	7.78 ± 0.09	7.49 ± 0.07	7.14 ± 0.44	7.23 ± 0.17	7.49 ± 0.13	7.20 ± 0.24	7.16 ± 0.11	7.18 ± 0.15
22:5n-3	5.12 ± 0.06 ^a^	5.21 ± 0.14 ^a^	5.72 ± 0.30 ^bc^	5.79 ± 0.07 ^bc^	5.50 ± 0.09 ^abc^	5.85 ± 0.16 ^c^	5.37 ± 0.12 ^ab^	6.45 ± 0.04 ^d^
22:6n-3	19.3 ± 0.53 ^f^	18.3 ± 0.06 ^ef^	17.2 ± 0.40 ^d^	15.6 ± 0.29 ^b^	17.5 ± 0.30 ^de^	16.1 ± 0.38 ^bc^	16.9 ± 0.19 ^cd^	13.3 ± 0.39 ^a^
∑n-3 PUFA	34.8 ± 0.54 ^f^	33.6 ± 0.07 ^ef^	32.6 ± 0.52 ^cde^	30.9 ± 0.39 ^b^	32.9 ± 0.32 ^de^	31.5 ± 0.50 ^bc^	31.9 ± 0.24 ^bcd^	29.1 ± 0.51 ^a^
DHA/EPA	2.48 ± 0.06 ^d^	2.45 ± 0.03 ^d^	2.42 ± 0.10 ^d^	2.16 ± 0.07 ^b^	2.33 ± 0.03 ^cd^	2.23 ± 0.03 ^bc^	2.36 ± 0.01 ^cd^	1.85 ± 0.05 ^a^
∑C18-PUFA	11.4 ± 0.11 ^ab^	11.3 ± 0.12 ^ab^	11.0 ± 0.50 ^a^	11.9 ± 0.30 ^bc^	11.5 ± 0.06 ^ab^	11.7 ± 0.14 ^ab^	11.4 ± 0.09 ^ab^	12.5 ± 0.32 ^c^
∑LC-PUFA	35.5 ± 0.53 ^f^	34.4 ± 0.11 ^ef^	33.3 ± 0.46 ^cde^	31.5 ± 0.41 ^b^	33.5 ± 0.28 ^de^	32.1 ± 0.49 ^bc^	32.5 ± 0.27 ^bcd^	29.7 ± 0.51 ^a^

Data in a same row not sharing the same superscript letter were significantly different (*p* < 0.05). TFA: total fatty acids; SFA: saturated fatty acid; MUFA: monounsaturated fatty acid; PUFA: polyunsaturated fatty acid, LC-PUFA: long-chain polyunsaturated fatty acids.

**Table 5:** Liver fatty acid composition of experimental tiger puffer (% TFA, mean ± standard error).

Fatty Acid	FO-C	GDR			AFG			BT-C
		25BT	50BT	75BT	1BT-1FO	2BT-1FO	3BT-1FO	
14:0	3.88 ± 0.15 ^d^	3.49 ± 0.09 ^cd^	3.29 ± 0.14 ^bc^	2.91 ± 0.12 ^ab^	3.40 ± 0.13 ^c^	3.13 ± 0.12 ^bc^	3.53 ± 0.12 ^cd^	2.73 ± 0.13 ^a^
16:0	18.9 ± 0.09	19.5 ± 0.10	19.1 ± 0.25	19.5 ± 0.20	19.9 ± 0.66	18.6 ± 1.09	19.0 ± 0.28	20.1 ± 0.43
18:0	7.74 ± 0.19 ^a^	9.06 ± 0.39 ^bc^	9.38 ± 0.17 ^bc^	9.88 ± 0.29 ^cd^	8.81 ± 0.29 ^abc^	8.80 ± 0.73 ^abc^	8.68 ± 0.12 ^ab^	10.9 ± 0.26 ^d^
∑SFA	30.5 ± 0.08 ^a^	32.1 ± 0.21 ^ab^	31.8 ± 0.21 ^ab^	32.3 ± 0.23 ^ab^	32.1 ± 0.81 ^ab^	30.5 ± 1.73 ^a^	31.2 ± 0.31 ^a^	33.8 ± 0.49 ^b^
16:1n-7	8.77 ± 0.05 ^d^	8.37 ± 0.32 ^cd^	8.42 ± 0.16 ^cd^	8.03 ± 0.24 ^bc^	8.11 ± 0.06 ^bcd^	7.65 ± 0.39 ^ab^	8.28 ± 0.11 ^bcd^	7.15 ± 0.13 ^a^
18:1n-9	23.7 ± 0.12 ^a^	25.0 ± 0.40 ^a^	29.0 ± 0.28 ^b^	32.6 ± 0.22 ^c^	28.5 ± 0.39 ^b^	28.2 ± 1.82 ^b^	27.81 ± 0.1 ^b^	35.9 ± 0.88 ^d^
∑MUFA	32.4 ± 0.09 ^a^	33.4 ± 0.22 ^ab^	37.4 ± 0.14 ^c^	40.6 ± 0.23 ^d^	36.6 ± 0.34 ^c^	35.9 ± 2.21 ^bc^	36.1 ± 0.09 ^c^	43.1 ± 0.83 ^d^
18:2n-6	12.5 ± 0.05 ^f^	11.8 ± 0.15 ^ef^	10.8 ± 0.11 ^cd^	9.49 ± 0.25 ^b^	10.8 ± 0.47 ^cd^	10.1 ± 0.44 ^bc^	11.3 ± 0.14 ^bde^	8.57 ± 0.06 ^a^
18:3n-6	0.22 ± 0.00 ^c^	0.20 ± 0.00 ^dc^	0.19 ± 0.01 ^cd^	0.16 ± 0.00 ^b^	0.17 ± 0.01 ^bc^	0.15 ± 0.01 ^b^	0.18 ± 0.01 ^bcd^	0.07 ± 0.01 ^a^
20:2n-6	0.77 ± 0.02 ^d^	0.77 ± 0.02 ^d^	0.72 ± 0.00 ^cd^	0.64 ± 0.01 ^bc^	0.70 ± 0.01 ^bcd^	0.63 ± 0.05 ^ab^	0.71 ± 0.01 ^bcd^	0.62 ± 0.02 ^a^
20:4n-6	0.09 ± 0.00 ^c^	0.09 ± 0.01 ^c^	0.08 ± 0.00 ^bc^	0.07 ± 0.00 ^ab^	0.08 ± 0.00 ^bc^	0.07 ± 0.01 ^ab^	0.08 ± 0.00 ^bc^	0.06 ± 0.00 ^a^
∑n-6 PUFA	13.5 ± 0.06 ^f^	12.8 ± 0.15 ^ef^	11.8 ± 0.12 ^d^	10.4 ± 0.27 ^b^	11.7 ± 0.49 ^cd^	10.9 ± 0.51 ^bc^	12.3 ± 0.16 ^de^	9.32 ± 0.05 ^a^
18:3n-3	1.76 ± 0.01 ^e^	1.65 ± 0.06 ^de^	1.42 ± 0.01 ^bc^	1.20 ± 0.03 ^b^	1.29 ± 0.13 ^b^	1.20 ± 0.12 ^b^	1.52 ± 0.04 ^cd^	0.82 ± 0.01 ^a^
20:3n-3	0.48 ± 0.03 ^bc^	0.47 ± 0.01 ^bc^	0.43 ± 0.01 ^ab^	0.38 ± 0.00 ^ab^	0.48 ± 0.05 ^bc^	0.49 ± 0.05 ^bc^	0.54 ± 0.04 ^c^	0.35 ± 0.04 ^a^
20:5n-3	6.25 ± 0.12 ^e^	5.59 ± 0.05 ^d^	4.77 ± 0.01 ^c^	3.89 ± 0.08 ^b^	4.82 ± 0.46 ^c^	4.05 ± 0.22 ^b^	5.21 ± 0.17 ^cd^	3.06 ± 0.10 ^a^
22:5n-3	4.04 ± 0.10 ^d^	3.83 ± 0.05 ^cd^	3.54 ± 0.08 ^bc^	3.35 ± 0.05 ^b^	3.64 ± 0.11 ^bc^	3.44 ± 0.20 ^b^	3.80 ± 0.12 ^cd^	2.77 ± 0.08 ^a^
22:6n-3	6.76 ± 0.10 ^e^	6.03 ± 0.14 ^d^	5.00 ± 0.04 ^c^	4.13 ± 0.03 ^a^	5.36 ± 0.16 ^c^	3.94 ± 0.45 ^a^	5.48 ± 0.15 ^cd^	2.83 ± 0.17 ^a^
∑n-3 PUFA	19.3 ± 0.08 ^e^	17.6 ± 0.19 ^d^	15.2 ± 0.11 ^c^	12.9 ± 0.16 ^b^	15.6 ± 0.83 ^c^	13.1 ± 0.89 ^b^	16.5 ± 0.33 ^cd^	9.82 ± 0.36 ^a^
DHA/EPA	1.08 ± 0.04 ^bc^	1.08 ± 0.03 ^bc^	1.05 ± 0.01 ^ab^	1.06 ± 0.01 ^ab^	1.13 ± 0.08 ^c^	0.97 ± 0.06 ^ab^	1.05 ± 0.03 ^ab^	0.92 ± 0.03 ^a^
∑C18-PUFA	14.4 ± 0.05 ^f^	13.6 ± 0.18 ^ef^	12.5 ± 0.12 ^cd^	10.9 ± 0.28 ^b^	12.3 ± 0.61 ^cd^	11.4 ± 0.57 ^bc^	13.0 ± 0.19 ^de^	9.46 ± 0.06 ^a^
∑LC-PUFA	18.4 ± 0.09 ^e^	16.8 ± 0.15 ^d^	14.5 ± 0.12 ^c^	12.4 ± 0.16 ^b^	15.1 ± 0.70 ^c^	12.6 ± 0.82 ^b^	15.8 ± 0.29 ^cd^	9.68 ± 0.32 ^a^

Data in a same row not sharing the same superscript letter were significantly different (*p* < 0.05). TFA: total fatty acids; SFA: saturated fatty acid; MUFA: monounsaturated fatty acid; PUFA: polyunsaturated fatty acid, LC-PUFA: long-chain polyunsaturated fatty acids.

**Table 6:** Intestine fatty acid composition of experimental tiger puffer (% TFA, mean ± standard error).

Fatty Acid	FO-C	GDR			AFG			BT-C
		25BT	50BT	75BT	1BT-1FO	2BT-1FO	3BT-1FO	
14:0	2.21 ± 0.05 ^b^	2.11 ± 0.16 ^b^	2.20 ± 0.08 ^b^	1.73 ± 0.06 ^a^	2.55 ± 0.12 ^c^	2.84 ± 0.14 ^c^	2.80 ± 0.05 ^c^	1.46 ± 0.12 ^a^
16:0	21.9 ± 0.32 ^ab^	22.5 ± 0.87 ^bc^	21.6 ± 0.39 ^ab^	20.5 ± 0.75 ^a^	22.9 ± 0.23 ^bc^	23.4 ± 0.19 ^c^	22.7 ± 0.60 ^c^	20.6 ± 0.33 ^ab^
18:0	14.2 ± 0.43 ^c^	14.0 ± 0.56 ^c^	14.4 ± 0.38 ^c^	16.1 ± 0.26 ^d^	13.4 ± 0.56 ^bc^	12.1 ± 0.41 ^ab^	11.9 ± 0.53 ^a^	16.4 ± 0.46 ^d^
∑SFA	38.3 ± 0.70	38.6 ± 1.27	38.3 ± 0.36	38.3 ± 0.54	38.9 ± 0.37	38.3 ± 0.45	37.4 ± 0.50	38.4 ± 0.31
16:1n-7	3.91 ± 0.20 ^b^	3.95 ± 0.18 ^b^	3.78 ± 0.12 ^b^	3.15 ± 0.27 ^a^	4.36 ± 0.29 ^bc^	5.01 ± 0.22 ^d^	4.79 ± 0.09 ^cd^	2.71 ± 0.14 ^a^
18:1n-9	16.8 ± 0.20 ^ab^	18.5 ± 0.62 ^cd^	18.6 ± 0.41 ^d^	18.7 ± 0.49 ^d^	15.5 ± 0.36 ^a^	17.2 ± 0.40 ^bc^	16.4 ± 0.32 ^ab^	20.6 ± 0.57 ^d^
∑MUFA	20.7 ± 0.36 ^ab^	22.4 ± 0.51 ^bc^	22.3 ± 0.53 ^bc^	21.9 ± 0.74 ^bc^	19.8 ± 0.65 ^a^	22.2 ± 0.62 ^bc^	21.2 ± 0.40 ^ab^	23.3 ± 0.71 ^c^
18:2n-6	12.2 ± 0.28 ^bc^	12.0 ± 0.53 ^ab^	12.0 ± 0.20 ^ab^	11.2 ± 0.08 ^a^	12.9 ± 0.14 ^cd^	13.1 ± 0.14 ^d^	13.2 ± 0.27 ^d^	11.2 ± 0.10 ^a^
18:3n-6	0.09 ± 0.01 ^d^	0.07 ± 0.01 ^bc^	0.09 ± 0.00 ^d^	0.04 ± 0.00 ^a^	0.12 ± 0.01 ^e^	0.13 ± 0.01 ^e^	0.08 ± 0.00 ^cd^	0.05 ± 0.01 ^ab^
20:2n-6	1.19 ± 0.02 ^b^	1.02 ± 0.09 ^ab^	0.72 ± 0.27 ^a^	0.97 ± 0.06 ^ab^	1.14 ± 0.04 ^b^	1.10 ± 0.05 ^b^	1.12 ± 0.04 ^b^	0.90 ± 0.02 ^ab^
20:4n-6	0.10 ± 0.01 ^a^	0.13 ± 0.00 ^ab^	0.13 ± 0.01 ^ab^	0.15 ± 0.02 ^b^	0.15 ± 0.01 ^b^	0.13 ± 0.01 ^a^	0.11 ± 0.01 ^ab^	0.11 ± 0.02 ^a^
∑n-6 PUFA	13.6 ± 0.28 ^cd^	13.2 ± 0.63 ^bc^	12.9 ± 0.13 ^abc^	12.4 ± 0.09 ^ab^	14.3 ± 0.12 ^d^	14.4 ± 0.19 ^d^	14.5 ± 0.30 ^d^	12.2 ± 0.08 ^a^
18:3n-3	0.64 ± 0.04 ^ab^	0.60 ± 0.07 ^ab^	0.63 ± 0.03 ^ab^	0.50 ± 0.03 ^a^	0.50 ± 0.22 ^a^	0.82 ± 0.02 ^b^	0.83 ± 0.04 ^b^	0.38 ± 0.03 ^a^
20:3n-3	2.06 ± 0.17	1.78 ± 0.14	1.86 ± 0.03	2.00 ± 0.23	1.94 ± 0.17	1.63 ± 0.05	1.72 ± 0.09	1.95 ± 0.16
20:5n-3	5.05 ± 0.14 ^ab^	4.47 ± 0.49 ^ab^	4.90 ± 0.25 ^ab^	4.57 ± 0.45 ^ab^	5.45 ± 0.23 ^c^	4.93 ± 0.15 ^ab^	5.29 ± 0.17 ^bc^	4.31 ± 0.05 ^a^
22:5n-3	3.82 ± 0.11 ^bc^	3.79 ± 0.04 ^bc^	3.90 ± 0.10 ^bc^	4.28 ± 0.22 ^cd^	4.01 ± 0.23 ^c^	3.26 ± 0.08 ^a^	3.44 ± 0.14 ^ab^	4.76 ± 0.25 ^d^
22:6n-3	10.6 ± 0.05 ^b^	9.95 ± 0.29 ^b^	10.0 ± 0.17 ^b^	10.3 ± 0.13 ^b^	10.0 ± 0.19 ^b^	9.07 ± 0.29 ^a^	10.2 ± 0.18 ^b^	8.75 ± 0.31 ^a^
∑n-3 PUFA	22.1 ± 0.29 ^b^	20.6 ± 0.93 ^ab^	21.3 ± 0.37 ^ab^	21.6 ± 1.03 ^ab^	21.9 ± 0.54 ^b^	19.7 ± 0.15 ^a^	21.5 ± 0.50 ^ab^	20.2 ± 0.69 ^ab^
DHA/EPA	2.10 ± 0.06 ^ab^	2.28 ± 0.22 ^ab^	2.05 ± 0.07 ^ab^	2.29 ± 0.22 ^b^	1.85 ± 0.11 ^a^	1.85 ± 0.11 ^a^	1.94 ± 0.05 ^ab^	2.03 ± 0.07 ^ab^
∑C18-PUFA	12.9 ± 0.32 ^b^	12.6 ± 0.61 ^b^	12.7 ± 0.21 ^b^	11.7 ± 0.05 ^a^	13.5 ± 0.09 ^bc^	14.0 ± 0.14 ^c^	14.1 ± 0.29 ^c^	11.6 ± 0.13 ^a^
∑LC-PUFA	22.8 ± 0.28 ^b^	21.1 ± 0.95 ^ab^	21.5 ± 0.56 ^ab^	22.2 ± 1.09 ^ab^	22.7 ± 0.43 ^b^	20.1 ± 0.19 ^a^	21.9 ± 0.48 ^ab^	20.8 ± 0.74 ^ab^

Data in a same row not sharing the same superscript letter were significantly different (*p* < 0.05). TFA: total fatty acids; SFA: saturated fatty acid; MUFA: monounsaturated fatty acid; PUFA: polyunsaturated fatty acid, LC-PUFA: long-chain polyunsaturated fatty acids.

**Table 7:** Relative mRNA expression of lipid metabolism genes in the liver of experimental fish (mean ± standard error).

Parameters	FO-C	GDR			AFG			BT-C
		25BT	50BT	75BT	1BT-1FO	2BT-1FO	3BT-1FO	
Lipogenesis								
*acacβ*	1.00 ± 0.14 ^ab^	1.11 ± 0.09 ^ab^	1.38 ± 0.21 ^b^	0.87 ± 0.13 ^a^	1.14 ± 0.13 ^ab^	0.81 ± 0.04 ^a^	1.12 ± 0.15 ^ab^	0.83 ± 0.08 ^a^
*fas*	1.00 ± 0.09 ^ab^	1.26 ± 0.16 ^abc^	1.41 ± 0.22 ^bc^	1.14 ± 0.16 ^ab^	1.01 ± 0.08 ^ab^	1.59 ± 0.11 ^c^	0.91 ± 0.04 ^a^	1.36 ± 0.13 ^abc^
*β*-oxidation								
*cpt-1*	1.00 ± 0.10 ^ab^	0.94 ± 0.15 ^ab^	0.97 ± 0.12 ^ab^	1.01 ± 0.22 ^ab^	0.98 ± 0.05 ^ab^	1.02 ± 0.10 ^ab^	0.64 ± 0.03 ^a^	1.11 ± 0.17 ^b^
*vlc*	1.00 ± 0.08 ^ab^	0.84 ± 0.03 ^a^	1.39 ± 0.15 ^abc^	2.35 ± 0.17 ^d^	1.53 ± 0.24 ^bc^	1.00 ± 0.21 ^ab^	0.89 ± 0.04 ^a^	1.68 ± 0.25 ^c^
*acox1*	1.00 ± 0.10 ^a^	1.23 ± 0.07 ^ab^	1.33 ± 0.15 ^b^	1.01 ± 0.08 ^a^	1.33 ± 0.03 ^b^	1.11 ± 0.01 ^ab^	0.95 ± 0.13 ^a^	1.18 ± 0.05 ^ab^
*acox3*	1.00 ± 0.04 ^a^	0.95 ± 0.09 ^a^	1.57 ± 0.12 ^c^	1.38 ± 0.1 b^c^	1.12 ± 0.03 ^ab^	1.18 ± 0.08 ^ab^	1.33 ± 0.10 ^bc^	1.85 ± 0.11 ^d^
*ehhadh*	1.00 ± 0.03 ^ab^	0.83 ± 0.09 ^a^	1.25 ± 0.14 ^bc^	1.36 ± 0.06 ^c^	0.83 ± 0.08 ^a^	0.98 ± 0.09 ^ab^	0.77 ± 0.09 ^a^	1.39 ± 0.08 ^c^
*acaa1*	1.00 ± 0.08 ^a^	1.54 ± 0.10 ^bc^	1.73 ± 0.06 ^c^	1.59 ± 0.05 ^c^	1.21 ± 0.24 ^ab^	1.43 ± 0.09 ^bc^	0.95 ± 0.06 ^a^	1.67 ± 0.12 ^c^
*acaa2*	1.00 ± 0.04 ^ab^	0.79 ± 0.08 ^a^	1.01 ± 0.03 ^ab^	1.14 ± 0.09 ^b^	1.20 ± 0.11 ^b^	1.02 ± 0.11 ^ab^	0.77 ± 0.10 ^a^	0.97 ± 0.11 ^ab^
Biosynthesis of glycerides								
*dgat1*	1.00 ± 0.06 ^bc^	1.30 ± 0.13 ^cd^	1.14 ± 0.08 ^cd^	0.97 ± 0.07 ^bc^	0.92 ± 0.06 ^bc^	0.79 ± 0.03 ^ab^	0.57 ± 0.04 ^a^	1.17 ± 0.10 ^cd^
*mgat*	1.00 ± 0.10 ^a^	1.03 ± 0.16 ^a^	0.97 ± 0.08 ^a^	0.99 ± 0.01 ^a^	0.92 ± 0.06 ^a^	1.43 ± 0.06 ^b^	1.09 ± 0.11 ^a^	0.91 ± 0.08 ^a^
Hydrolysis of glycerides								
*mgll*	1.00 ± 0.06 ^a^	1.26 ± 0.14 ^a^	1.94 ± 0.19 ^b^	1.34 ± 0.11 ^a^	1.31 ± 0.13 ^a^	3.28 ± 0.04 ^c^	3.38 ± 0.10 ^c^	1.11 ± 0.14 ^a^
*hsl*	1.00 ± 0.09 ^d^	0.70 ± 0.07 ^ab^	0.91 ± 0.05 ^c^	0.92 ± 0.06 ^bc^	0.83 ± 0.09 ^c^	0.63 ± 0.03 ^a^	0.78 ± 0.01 ^abc^	1.12 ± 0.04 ^d^
*atgl*	1.00 ± 0.07 ^bc^	0.67 ± 0.06 ^a^	1.00 ± 0.09 ^bc^	1.25 ± 0.05 ^d^	1.23 ± 0.08 ^d^	0.99 ± 0.06 ^bc^	0.89 ± 0.08 ^b^	1.15 ± 0.06 ^cd^
*daglα*	1.00 ± 0.04 ^c^	0.58 ± 0.03 ^a^	0.66 ± 0.05 ^ab^	0.97 ± 0.02 ^c^	0.84 ± 0.12 ^bc^	0.66 ± 0.05 ^ab^	0.98 ± 0.09 ^c^	0.72 ± 0.05 ^ab^
Lipid digestion								
*bsal*	1.00 ± 0.07 ^c^	1.00 ± 0.10 ^c^	0.95 ± 0.05 ^bc^	0.70 ± 0.06 ^a^	1.47 ± 0.06 ^c^	1.70 ± 0.10 ^d^	0.86 ± 0.05 ^abc^	0.75 ± 0.01 ^ab^
*lp*	1.00 ± 0.04 ^bc^	1.11 ± 0.13 ^bc^	0.72 ± 0.07 ^a^	0.57 ± 0.06 ^a^	0.75 ± 0.05 ^ab^	0.76 ± 0.12 ^ab^	0.82 ± 0.07 ^a^	0.70 ± 0.05 ^a^
Lipid transportation								
*fatp1*	1.00 ± 0.07 ^bc^	1.03 ± 0.07 ^bc^	1.15 ± 0.04 ^c^	1.01 ± 0.03 ^bc^	0.99 ± 0.10 ^bc^	0.75 ± 0.03 ^a^	0.79 ± 0.08 ^a^	0.89 ± 0.04 ^ab^
*apoa1*	1.00 ± 0.10 ^ab^	1.28 ± 0.10 ^bc^	1.85 ± 0.06 ^d^	0.99 ± 0.04 ^ab^	1.39 ± 0.12 ^c^	1.21 ± 0.13 ^bc^	0.86 ± 0.09 ^a^	1.31 ± 0.12 ^bc^
*lpl*	1.00 ± 0.07 ^bc^	1.35 ± 0.10 ^d^	1.19 ± 0.04 ^cd^	0.76 ± 0.06 ^a^	0.78 ± 0.03 ^a^	0.96 ± 0.09 ^ab^	1.01 ± 0.05 ^bc^	1.04 ± 0.04 ^bc^
*fabp1*	1.00 ± 0.03 ^b^	0.84 ± 0.13 ^b^	1.18 ± 0.09 ^d^	1.39 ± 0.04 ^c^	0.58 ± 0.01 ^a^	0.68 ± 0.04 ^ab^	1.48 ± 0.01 ^cd^	1.61 ± 0.08 ^d^
*apoa4*	1.00 ± 0.11 ^bc^	1.07 ± 0.03 ^c^	1.15 ± 0.08 ^c^	0.60 ± 0.05 ^a^	1.00 ± 0.05 ^bc^	1.66 ± 0.07 ^d^	1.22 ± 0.06 ^c^	0.84 ± 0.06 ^b^
*apob100*	1.00 ± 0.05 ^ab^	0.93 ± 0.04 ^ab^	1.06 ± 0.09 ^bc^	0.99 ± 0.05 ^ab^	0.89 ± 0.10 ^ab^	0.81 ± 0.07 ^a^	0.94 ± 0.03 ^ab^	1.16 ± 0.06 ^c^
*apoe1*	1.00 ± 0.01 ^c^	0.99 ± 0.07 ^c^	0.78 ± 0.02 ^ab^	0.72 ± 0.07 ^a^	0.95 ± 0.03 ^bc^	1.06 ± 0.11 ^c^	1.40 ± 0.07 ^d^	0.76 ± 0.06 ^ab^
*lipc*	1.00 ± 0.02 ^a^	1.10 ± 0.15 ^ab^	1.39 ± 0.13 ^bc^	1.61 ± 0.14 ^c^	1.46 ± 0.10 ^bc^	1.73 ± 0.12 ^cd^	1.82 ± 0.03 ^d^	2.25 ± 0.04 ^e^
*mttp*	1.00 ± 0.02 ^bc^	0.80 ± 0.06 ^ab^	1.02 ± 0.08 ^bc^	0.78 ± 0.02 ^ab^	0.64 ± 0.07 ^a^	0.75 ± 0.07 ^ab^	0.93 ± 0.02 ^b^	1.17 ± 0.09 ^c^
*fabp10a*	1.00 ± 0.09 ^c^	0.66 ± 0.05 ^bc^	0.37 ± 0.01 ^a^	0.51 ± 0.03 ^ab^	0.39 ± 0.05 ^a^	0.45 ± 0.07 ^ab^	0.46 ± 0.01 ^ab^	0.63 ± 0.08 ^b^
Lipid metabolism-related transcriptional factors								
*srebf1*	1.00 ± 0.08 ^a^	1.66 ± 0.17 ^c^	1.34 ± 0.18 ^ab^	1.01 ± 0.10 ^a^	1.69 ± 0.09 ^c^	1.47 ± 0.05 ^bc^	1.56 ± 0.04 ^c^	1.16 ± 0.06 ^ab^
*pparα1*	1.00 ± 0.08 ^b^	1.29 ± 0.11 ^c^	0.82 ± 0.03 ^ab^	0.97 ± 0.10 ^b^	0.87 ± 0.07 ^ab^	0.70 ± 0.05 ^a^	0.72 ± 0.09 ^a^	0.76 ± 0.01 ^a^
*lxrα*	1.00 ± 0.03 ^bc^	0.74 ± 0.02 ^ab^	0.87 ± 0.07 ^b^	1.21 ± 0.04 ^c^	0.63 ± 0.04 ^a^	0.75 ± 0.07 ^ab^	0.67 ± 0.04 ^a^	0.94 ± 0.09 ^b^
*pparγ*	1.00 ± 0.06 ^a^	1.18 ± 0.07 ^ab^	1.26 ± 0.07 ^b^	1.38 ± 0.06 ^bc^	1.14 ± 0.05 ^ab^	1.25 ± 0.13 ^b^	1.56 ± 0.06 ^c^	0.98 ± 0.05 ^a^
*pparβ*	1.00 ± 0.06 ^ab^	0.83 ± 0.08 ^a^	0.88 ± 0.11 ^a^	0.99 ± 0.09 ^ab^	0.75 ± 0.05 ^a^	0.75 ± 0.08 ^a^	1.20 ± 0.06 ^bc^	1.42 ± 0.09 ^c^
*pparα2*	1.00 ± 0.03 ^b^	0.82 ± 0.08 ^a^	0.75 ± 0.07 ^a^	0.87 ± 0.06 ^a^	0.69 ± 0.05 ^a^	0.83 ± 0.09 ^a^	0.66 ± 0.02 ^a^	0.84 ± 0.08 ^a^
*fxr*	1.00 ± 0.04 ^a^	1.66 ± 0.14 ^c^	1.44 ± 0.07 ^b^	1.43 ± 0.01 ^b^	1.70 ± 0.02 ^c^	1.52 ± 0.03 ^bc^	0.89 ± 0.06 ^a^	1.34 ± 0.08 ^b^
*lrh-1*	1.00 ± 0.01 ^bc^	1.09 ± 0.05 ^c^	1.00 ± 0.02 ^bc^	0.99 ± 0.05 ^bc^	1.00 ± 0.03 ^bc^	0.90 ± 0.01 ^b^	0.65 ± 0.03 ^a^	1.06 ± 0.04 ^c^
*hnf4α*	1.00 ± 0.05 ^b^	0.76 ± 0.06 ^ab^	0.91 ± 0.06 ^b^	0.91 ± 0.00 ^b^	0.93 ± 0.04 ^b^	1.00 ± 0.08 ^b^	0.58 ± 0.04 ^a^	1.10 ± 0.06 ^b^
Cholesterol and bile acid biosynthesis								
*hmgcr*	1.00 ± 0.08 ^a^	2.15 ± 0.13 ^bc^	2.17 ± 0.18 ^bc^	2.01 ± 0.07 ^b^	1.81 ± 0.15 ^b^	2.10 ± 0.13 ^bc^	2.46 ± 0.18 ^c^	1.11 ± 0.13 ^a^
*cyp7a1*	1.00 ± 0.10 ^bc^	0.73 ± 0.19 ^ab^	0.59 ± 0.04 ^a^	0.70 ± 0.04 ^ab^	0.44 ± 0.10 ^a^	1.08 ± 0.16 ^c^	0.60 ± 0.06 ^a^	0.73 ± 0.03 ^ab^

Data in a same row not sharing the same superscript letter were significantly different (*p* < 0.05).

## Data Availability

The data presented in this study are available in the manuscript.

## Supplementary Materials

The following supporting information can be downloaded at: https://www.mdpi.com/article/10.3390/ani13193037/s1, Table S1: Sequences of the primers for lipid metabolism genes and muscle MtDNA genes studied in this work; Table S2: Growth performance of experimental tiger puffer (mean ± standard error); Table S3: Lipid metabolism-related biochemical parameters in the serum (mean ± standard error); Figure S1: Relative abundance of main fatty acids in experimental diets. The fatty acid contents in group FO-C were normalized to be 1; Figure S2: Relative abundance of main fatty acids in the muscle of juvenile tiger puffer. The fatty acid contents in group FO-C were normalized to be 1; Figure S3: Relative abundance of main fatty acids in the liver of juvenile tiger puffer. The fatty acid contents in group FO-C were normalized to be 1.

## Abbreviations

Abbreviation	Full name
Non-gene name	
FO	fish oil
BT	beef tallow
GDR	Graded Dietary Replacement of FO with BT
AFD	Alternate Feeding between FO- and BT-based Diets
LC-PUFA	long-chain polyunsaturated fatty acids
SFA	saturated fatty acid
MUFA	monounsaturated fatty acid
PUFA	polyunsaturated fatty acid
TFA	total fatty acid
DHA	docosahexaenoic acid
EPA	eicosapentaenoic acid
HSI	hepatosomatic index
VSI	visecrosomatic index
CF	condition factor
WG	weight gain
FBW	final body weight
FER	feed efficiency ratio
TG	triacylglycerol
TC	total cholesterol
TBA	total bile acid
HDL-C	high-density lipoprotein cholesterol
LDL-C	low-density lipoprotein cholesterol
CYTB	cytochrome B
Gene name	
*acacβ*	acetyl-CoA carboxylase beta
*fas*	fatty acid synthase
*cpt-1*	carnitine O-palmitoyltransferase-1
*vlcs*	very long-chain acyl-CoA synthetase
*acox1*	acyl-CoA oxidase 1, palmitoyl
*acox3*	acyl-CoA oxidase 3, pristanoyl
*ehhadh*	enoyl-CoA hydratase and 3-hydroxyacyl CoA dehydrogenase
*acaa*	acetyl-CoA acyltransferase
*dgat* *1*	diacylglycerol O-acyltransferase 1
*mgat2a*	2-acylglycerol O-acyltransferase 2-A-like (LOC101069338)
*atgl*	adipose triglyceride lipase (patatin like phospholipase domain containing 2 (*pnpla2*))
*dagl* *α*	diacylglycerol lipase, alpha
*hsl*	hormone-sensitive lipase
*mgll*	monoglyceride lipase
*bsal*	bile acid activated lipase
*lp*	inactive pancreatic lipase-related protein 1-like (LOC101064949)
*lpl*	lipoprotein lipase
*lipc*	lipase, hepatic
*fabp*	fatty acid binding protein
*fatp*	fatty acid transport protein (solute carrier family 27 member 1 (*slc27a1*))
*apo*	apolipoprotein
*mttp*	microsomal triglyceride transfer protein
*srebf* *1*	sterol regulatory element binding transcription factor 1
*ppar*	peroxisome proliferators-activated receptor
*fxr*	farnesoid X receptor (nuclear receptor subfamily 1, group H, member 4, *nr1h4*)
*lxr* *α*	liver X receptor alpha (nuclear receptor subfamily 1, group H, member 3, *nr1h3*)
*hnf* *4α*	hepatocyte nuclear factor 4, alpha
*lrh* *-1*	liver receptor homolog-1 (nuclear receptor subfamily 5, group A, member 2, *nr5a2*)
*hmgcr*	3-hydroxy-3-methylglutaryl-CoA reductase
*cyp7a1*	cholesterol 7-alpha-hydroxylase (cytochrome P450 7A1)
