# Supplementary material for: Feeding Strategy to Use Beef Tallow and Modify Farmed Tiger Puffer Fatty Acid Composition
Source: Animals (Basel). 2023 Sep 27;13(19):3037. doi: 10.3390/ani13193037 (PMC10571522; doi:10.3390/ani13193037)
Supplement: Supplementary file 1 [file animals-13-03037-s001.zip › animals-2604318-supplementary.pdf]

Supplementary Table S1 Primers information for the qPCR study on mtDNA and lipid metabolism-related genes in this work

Primer	Sequence (5'-3')	GenBank reference	PL (bp)
	mtDNA		
16S rRNA-F	ATGTGGACCTGTATGAATGGC	NC_004299.1	119
16S rRNA-R	CTCCATAGGGTCTTCTCGTCTT		
CTYB-F	CCTCCTGGGCTTCACAATCA	NC_004299.1	123
CTYB-R	TTAATGTGGGCGGGGGTAAC		
	lipid metabolism gene		
Lipogenesis			
acacβ-F	GAAAGGTTTGCTGTGCGACTA	XM_011615767.1	154
acacβ-R	TTACATCAGCGACCATTTCAGT		
fas-F	CTTTGCCGCTGTCATTCG	XM_011619859.1	78
fas-R	TGTCTCAACCCATTTGTAGTCG		
β-oxidation			
cpt-1-F	GGGGTTTGTGGTCAAGTTAGG	XM_011607269.1	186
cpt-1-R	ATAGATCCGTGGCGCTCAT		
vlc-qF	CGCTGTTCTTGGTGTTGGAC	XM_003969871.3	276
vlc-qR	GAGATTTGCTGCGGATGTTG		
acox1-qF	GCACGGCATCGCAAGTAAC	XM_029850253.1	145
acox1-qR	GAGATCGAAGGCATCCACC		
acox3-qF	GACTGTGGCTATCCGCTTCT	XM_029839734.1	214
acox3-qR	TTCCTGTCGGTCACTCTTGT		
ehhadh-qF	GGCACAATGGGAAGAGGCATT	XM_003961946.3	185
ehhadh-qR	TGGACGGTTTCGCTGTAGGTA		
acaa1-qF	GGACAACAGCAAAGCAAGAG	XM_029849183.1	110
acaa1-qR	ACCAGAAAAAACAGCCAAAA		
acaa2-qF	ACGGGGGTGTTTTGAAGGA	XM_003975006.3	159

acaa2-qR	CATGACGGGCAATGTAGGG		
dgat1-F	TGGTTTGTGAGCCGTTTCC	XM_003969352.2	185
dgat1-R	CTGGCATTCGTTTGACTTCG		
mgat2a-F	AAAGGCTTCATTAAATTGGC	XM_003978609.3	223
mgat2a-R	TGATGGCTTGTCTGTAGGG		
Hydrolysis of glycerides			
atgl-F	CCAACCTCTACAGGGTCTCA	XM_003967696.3	119
atgl-R	GTTTAGCAGCCCGTTCTTC		
daglα-F	CTGTTGGTGGAGTTGGTGTATG	XM_011610175.1	72
daglα-R	ATCAGAGCACGGCTGGTAAT		
hsl-F	CTCTTGCTATCGGTCTTGTGG	XM_011621066.1	113
hsl-R	TTCTGGGTCAATGGCATACTT		
mgll-F	CCATCCAGTCAAAGTGGGTCT	XM_003963030.2	110
mgll-R	CATCAGCTGCATGCCGAA		
Lipid digestion			
bsal-F	TTGAAGATGACTGACCCCGA	XM_003978375.2	162
bsal-R	GATGTCTGCTGCGTTGTGAA		
lp-F	CGTTTTCTCCTGTTCACCC	XM_029832009.1	97
lp-R	GACTCGTCCTCATCCCACT		
Lipid transport			
lpl-F	AGGGTCCACATCCGCAAA	NM_001305600.1	157
lpl-R	GTTTCTCCTTGCGGCTCAT		
lipc-F	GCGGCTTCAACAGCAGTAA	XM_011610357.1	215
lipc-R	GAGGTGCGCTATGTCTTTCC		
fabp1-F	CCATCGGTCTCCCTGATGAAG	XM_003974807.3	121
fabp1-R	TTGACCGTTACCTTCGGTCC		
fabp10a-F	CTGTGACCAACTCCTTTACCAT	XM_003965635.3	150
fabp10a-R	TCTCTCCACCTTTGAGCTCCTG		

fatp1-F	ATTGCAGACACCACAGGGAG	XM_003964742.3	219
fatp1-R	ATATCGTGACGCTCGTGCAT		
apoa1-F	CGATGACGCCGAGTACAAA	AB183289.1	104
apoa1-R	CGGTTATGGGAGAAACGCTA		
apoa4-F	TGCTTTCTGGGACTATGTTGC	NM_001078591.1	124
apoa4-R	GTTGACTTTGTCGGCACTCTC		
apob100-F	AGGGACATAGTCAAACCAAGGA	XM_011619944.1	127
apob100-R	AGAACACGAAGGCTGGACAC		
apoe1-F	TATTCAGACCCGCACCTCA	NM_001078592.1	201
apoe1-R	ATTTCCTCCATCTTGTCCTCC		
mttp-F	ATGCTAAGGGTCTGGTTCTGC	XM_011612378.1	124
mttp-R	ATGTCAGTGCTGCCGATCTT		
Lipid metabolism-related transcriptional factors			
srebf1-F	TTTCAGCATCCCACCTTCC	XM_011603881.1	158
srebf1-R	GGTGAACCGTGAGGACAACTA		
pparα1-F	TCAGTAGTTTATGGGTTGGTGG	NM_001097630.1	119
pparα1-R	GCGTGGACTCCGTAGTGGTA		
pparα2-F	CCAGAAGAAGAACCGCAACA	NM_001097629.1	149
pparα2-R	CCTCTTTCTCCACCATCTTGTT		
pparβ-F	AGCTGGAATACGACCGATGT	AB275887.1	249
pparβ-R	TCTTCAGGTAGGCGGAGTTG		
pparγ-F	CGCTGTCCCGACATCTGTAT	NM_001097627.1	146
pparγ-R	GAACTGCTCGCCTTCCATT		
fxr-F	GTGAACGACCACAAGTTTACCC	XM_003967283.2	166
fxr-R	AGACCAACAGATTACACCGGAT		
lxrα-F	GTGACGCACCACTAACAGCA	XM_011609917.1	191
lxrα-R	CTGACAACACCGAGCAAGACT		
hnf4α-F	GAGCCACGGGCAAACACTA	XM_011619034.1	199

hnf4α-R	AGGGTCCTACCTTCTTTCTTCAT		
lrh-1-F	CGCTGACATGCTGCCTAAA	XM_003974281.2	140
lrh-1-R	TCTCGTCCAAGTCTTCGTCAT		
Cholesterol and bile acid biosynthesis			
hmgcr-F	GCTGCTGGCAATCAAGTACAT	XM_003974466.2	143
hmgcr-R	AAACATACAACTCCTTCCTACAGC		
cyp7a1-F	CCTACCTGCTACCTTCTGGAGT	XM_003975521.2	237
cyp7a1-R	TCCTCTTTGGCAACACGAA		
Reference gene			
β-actin-F	GAGAGGGAAATCGTGCGTGA	XM_003964421.3	186
β-actin-R	GAAGGATGGCTGGAAGAGGG		
ef1α-F	TTGGAGGCATTGGAACTGT	NM_001037873.1	86
ef1α-R	GTTGACGGGAGCAAAGGT		
Reference gene			
β-actin-F	GACGCAAAACCTCCGAACTG	Gene ID 101079312	129
β-actin-R	CCTCCAAACGGATCAGCACA		
EF1α-F	TGGCCTTTAGCCGAATGAGG	Gene ID 653026	117
EF1α-R	TGTCGGGCCAATCAATCCAG		

PL: product length; *acacβ*: acetyl-CoA carboxylase beta; *fas*: fatty acid synthase; *cpt-1*: carnitine O-palmitoyltransferase-1; *vlcs*: very long-chain acyl-CoA synthetase; *acox1*: acyl-CoA oxidase 1, palmitoyl; *acox3*: acyl-CoA oxidase 3, pristanoyl; *ehhadh*: enoyl-CoA hydratase and 3-hydroxyacyl CoA dehydrogenase; *acaa*: acetyl-CoA acyltransferase; *dgat1*: diacylglycerol O-acyltransferase 1; *mgat2a*: 2-acylglycerol O-acyltransferase 2-A-like (LOC101069338); *atgl*: adipose triglyceride lipase (patatin like phospholipase domain containing 2 (*pnpla2*)); *dagla*: diacylglycerol lipase, alpha; *hsl*: hormone-sensitive lipase; *mgll*: monoglyceride lipase; *bsal*: bile acid activated lipase; *lp*: inactive pancreatic lipase-related protein 1-like (LOC101064949); *lpl*: lipoprotein lipase; *lipc*: lipase, hepatic; *fabp*: fatty acid binding protein; *fatp*: fatty acid transport protein (solute carrier family 27 member 1 (*slc27a1*)); *apo*: apolipoprotein; *mttp*: microsomal triglyceride transfer protein; *srebf1*: sterol regulatory element binding

transcription factor 1; *ppar*: peroxisome proliferators-activated receptor; *fxr*: farnesoid X receptor (nuclear receptor subfamily 1, group H, member 4, *nr1h4*); *lxrα*: liver X receptor alpha (nuclear receptor subfamily 1, group H, member 3, *nr1h3*); *hnf4α*: hepatocyte nuclear factor 4, alpha; *lrh-1*: liver receptor homolog-1 (nuclear receptor subfamily 5, group A, member 2, *nr5a2*); *hmgcr*: 3-hydroxy-3-methylglutaryl-CoA reductase; *cyp7a1*: cholesterol 7-alpha-hydroxylase (cytochrome P450 7A1); CYTB: cytochrome B.

Supplementary Table S2 Growth performance of experimental tiger puffer (mean ± standard error)

Parameters	FO-C	Graded FO replacement with BT			Alternate feeding between FO and BT			BT-C
		25%	50%	75%	1BT-1FO	2BT-1FO	3BT-1FO	
FBW (g)	75.5±1.36^{ab}	68.9±1.07^{a}	68.7±0.67^{a}	83.5±8.02^{b}	70.1±3.31^{a}	70.5±1.33^{a}	74.0±3.10^{ab}	75.6±4.26^{ab}
FER	0.71±0.03^{bc}	0.54±0.03^{bc}	0.36±0.09^{a}	0.78±0.18^{c}	0.62±0.03^{abc}	0.43±0.14^{ab}	0.61±0.05^{abc}	0.65±0.06^{abc}
WG (%)	628±11.3^{a}	574±8.89^{ab}	572±5.62^{a}	696±66.9^{b}	583±27.6^{a}	587±11.1^{a}	616±25.8^{ab}	630±35.5^{ab}
Survival (%)	59.1±4.76^{bc}	40.0±0.00^{ab}	32.4±6.87^{a}	48.6±1.65^{bc}	38.1±0.95^{ab}	30.5±5.30^{a}	45.7±4.36^{abc}	50.5±8.30^{bc}

Data in a same row sharing same superscript letters are not significantly different (*P*>0.05). FBW: final body weight; FER: feed efficiency ratio; WG: weight gain.

Supplementary Table S3 Lipid metabolism-related biochemical parameters in the serum (mean ± standard error)

Parameters	FO-C	Graded FO replacement with BT			Alternate feeding between FO and BT			BT-C
		25%	50%	75%	1BT-1FO	2BT-1FO	3BT-1FO	
TG (mmol L^{-1})	0.76±0.02^{d}	0.58±0.02^{b}	0.58±0.00^{b}	0.58±0.02^{b}	0.65±0.02^{c}	0.50±0.00^{a}	0.55±0.01^{b}	0.57±0.01^{b}
HDL-C (mmol L^{-1})	0.77±0.10^{bc}	1.05±0.15^{d}	0.37±0.01^{ab}	0.29±0.04^{a}	0.58±0.10^{b}	0.13±0.01^{a}	0.64±0.09^{b}	0.92±0.04^{cd}
LDL-C (mmol L^{-1})	3.08±0.10^{ab}	2.97±0.09^{a}	4.31±0.27^{c}	3.27±0.20^{ab}	3.01±0.13^{ab}	3.53±0.20^{b}	3.21±0.12^{ab}	3.12±0.09^{ab}
TBA (μmol L^{-1})	0.46±0.03^{a}	0.49±0.03^{a}	0.64±0.01^{b}	0.53±0.03^{ab}	0.57±0.04^{ab}	0.57±0.04^{ab}	0.60±0.05^{b}	0.53±0.04^{ab}
TC (mmol L^{-1})	4.83±0.20^{ab}	4.72±0.18^{ab}	5.30±0.29^{b}	4.17±0.26^{a}	4.31±0.19^{a}	4.25±0.19^{a}	4.42±0.08^{a}	4.83±0.07^{ab}

Data in a same row sharing same superscript letters are not significantly different (P>0.05). TG: triacylglycerol; HDL-C: high-density lipoprotein cholesterol; LDL-C: low-density lipoprotein cholesterol; TBA: total bile acid; TC: total cholesterol. The concentration of these parameters in serum were measured with commercial kits supplied by Nanjing Jiancheng Bioengineering Institute (Nanjing, China).

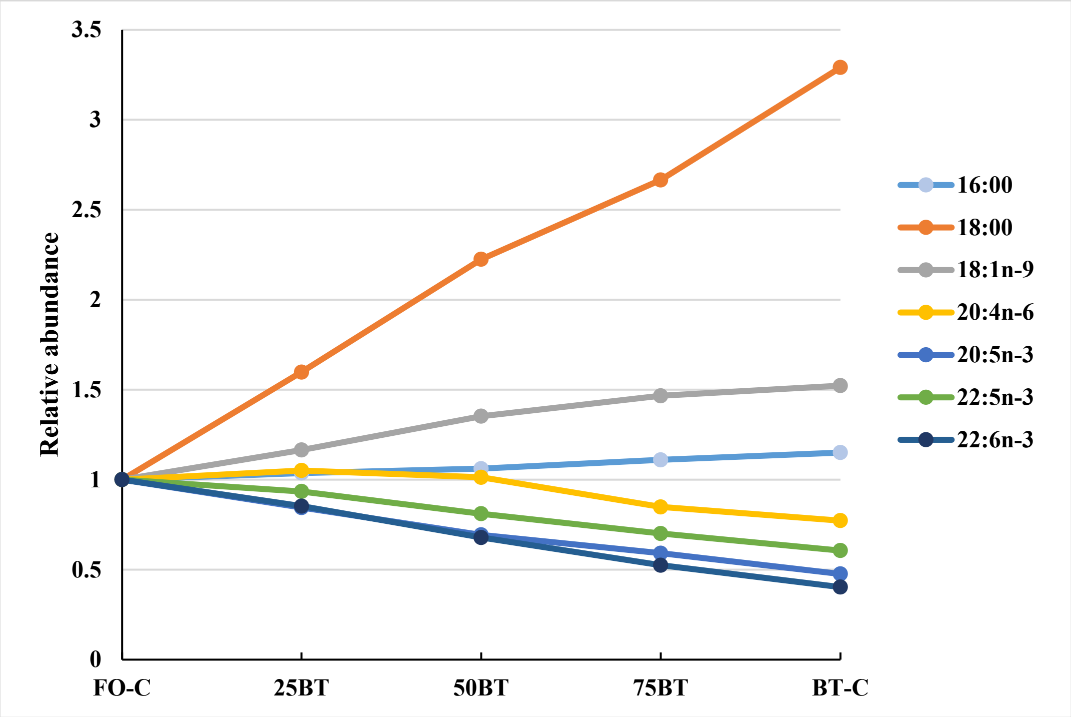

Supplementary Figure S1 Relative abundance of main fatty acids in experimental diets. The fatty acid contents in group FO-C were normalized to be 1.

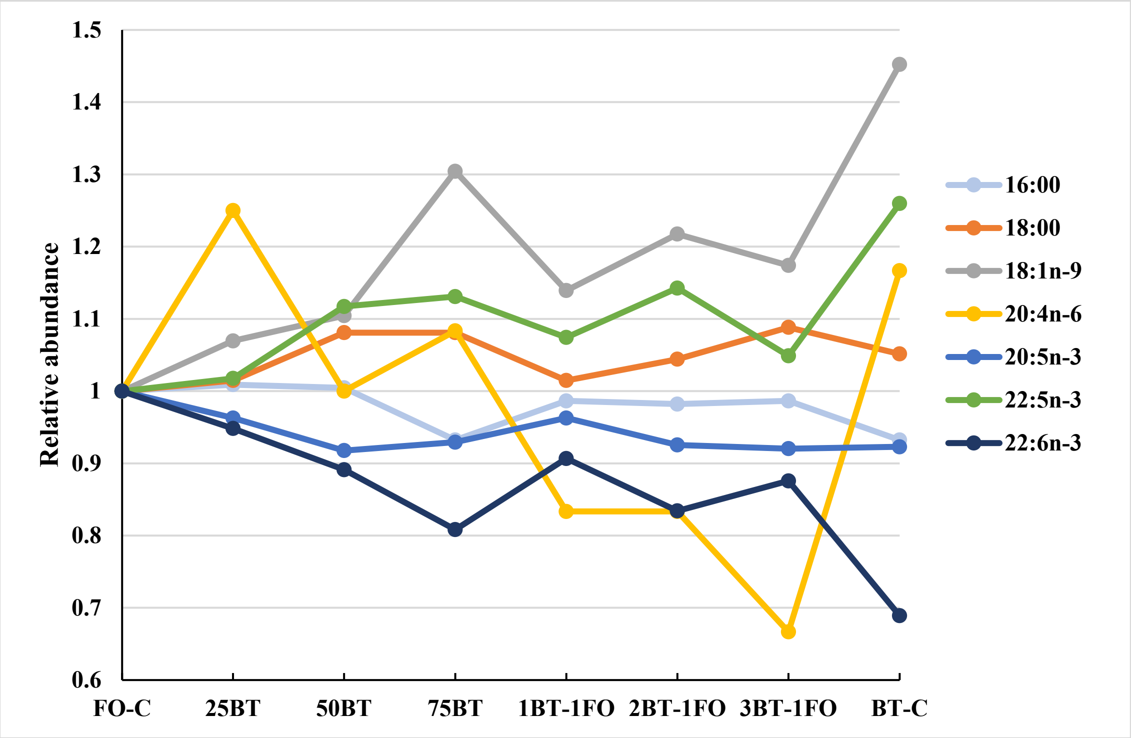

Supplementary Figure S2 Relative abundance of main fatty acids in the muscle of juvenile tiger puffer. The fatty acid contents in group FO-C were normalized to be 1.

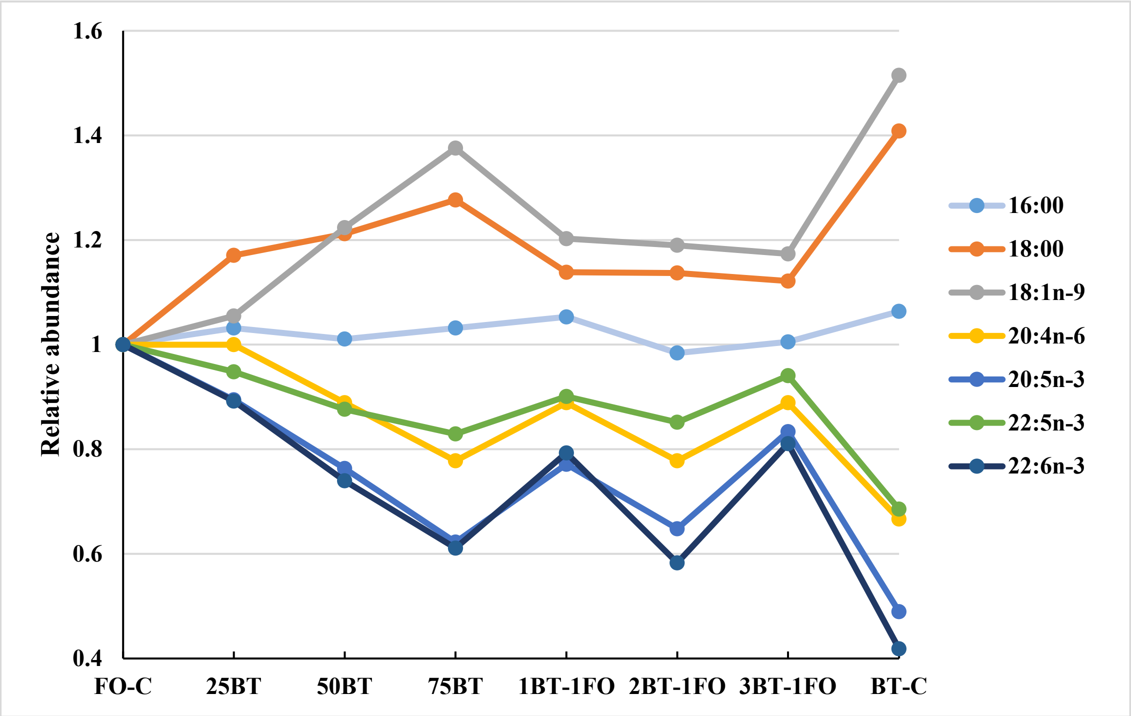

Supplementary Figure S3 Relative abundance of main fatty acids in the liver of juvenile tiger puffer. The fatty acid contents in group FO-C were normalized to be 1.
